# The Organization of the Southern Dental Association

**Published:** 1869-09

**Authors:** 


### EDITORIAL DEPARTMENT.

*The Organization of the Southern Dental Association.*—It is with great pleasure and no little pride that we are able to inform our readers that the first meeting of this new Association, organized for the benefit of Southern Dentists, which was held at Atlanta, Georgia, during the last week of July, was all that its warmest friends could have desired.

We have attended many assemblies of this character, but remember none which gave such universal satisfaction to those present as this the first meeting of the Southern Dental Association—a happy augury for its future usefulness.

Such was the harmony and pleasure experienced, that all who attended returned to their respective homes feeling satisfied that the sacrifice they had made in leaving their professional duties had not been too great. We also refer with pride to the fact that not an unpleasant word was spoken in the debates upon organization, modes of practice, &c., to interrupt the harmony of the meeting, a truly brotherly feeling prevailing altogether free from the jealousies so common to professional organizations. There was manifested on the part of every member present a willingness to do his part towards advancing the interests of the profession South, regardless of self aggrandizement.

We must congratulate the Association on the manner in which its several meetings were presided over, and the ability for conducting such business as was shown by all the officers appointed to the different chairs. The character of the members present would reflect honor upon any Association, and the universal good feeling which prevailed, together with the interest manifested in the proceedings, are in the highest degree encouraging for the future prosperity and permanence of this organization.

The number present at the organization of this Association is also a matter for congratulation, as no other Dental Association has ever been instituted with even half the number who participated in the Atlanta meeting. The Association was also happy in the choice of Atlanta as the place of its first meeting, for more *true hospitality* has never been extended to any professional gathering, than that which its members experienced from the citizens of this enterprising Southern City. The City Council of Atlanta, besides the free use of the City Hall, provided a sumptuous dinner for the members of the Association at the National Hotel, which was highly appreciated—it was such a repast as fully established the reputation of the proprietor of this first class house. A number of ladies graced the table with their presence, and several members of the City Council and the Medical Faculty of Atlanta. Among the guests present we were pleased to meet Prof. J. P. Logan, late of the Washington University of Medicine, Baltimore, but now practicing in Atlanta. The rail-road companies also extended courtesies and aid, which were duly appreciated. To Mr. Saml. Hape the proprietor of the Dental Depot, and to the members of the dental profession residing in Atlanta, the Association is under many obligations for the kindness and courtesies extended, and their labors to render the meeting in every way a pleasant one.

The good effects of this Atlanta meeting were shown by the organization of several State societies in States where no such action had before been taken. These State societies were organized immediately after the adjournment of the Association by prominent members in attendance; and this action clearly proves the justness of the statement we made when advocating the formation of the "Southern Dental Association" in the Journal, namely—that it would exercise such an influence for good as no one of the already existing Associations could accomplish.

